# Comparative Profiling of the Fecal Bacteriome, Mycobiome, and Protist Community in Wild Versus Captive (*Cervus canadensis*)

**DOI:** 10.3390/ani16010044

**Published:** 2025-12-24

**Authors:** Yalin Zhou, Yan Wu, Cuiliu Ma, Xingzhou Ruan, Muha Cha, Yulei Zhou, Tao Li, Weili Sun, Hanlu Liu

**Affiliations:** 1 College of Agriculture, Chifeng University, Chifeng 024000, China; 2 College of Animal Science and Technology, Qingdao Agricultural University, Qingdao 266109, China; 3 Gaogesitai Hanwula National Nature Reserve, Chifeng 024000, China; 4 Shanghai Veterinary Research Institute, Chinese Academy of Agricultural Sciences (CAAS), Shanghai 200241, China

**Keywords:** wapiti, wild, domestication, gut microbiome

## Abstract

Diet and living environments exert a profound influence on gut microbiota composition. This study presents the first comprehensive characterization of fecal bacteria, fungi, and protozoa in wild (WA) (*n* = 10) and captive (DA) (*n* = 11) wapiti (Cervus canadensis) in China. Results reveal distinct microbial profiles between the two groups. In wild wapiti, *Escherichia-Shigella* and *UCG-005* were the dominant bacterial genera, while *Succinivibrio* and *Treponema* predominated in captive individuals. Among fungi, *Agaricus* and *Preussia* were most abundant in wild wapiti, whereas *Xeromyces* was identified in captive ones. For protozoa, *Heteromita* was the primary genus in wild wapiti, while *Heteromita*, *Entamoeba*, and *Eimeria* were the main genera in captive wapiti. Functional predictions further underscored these differences. In wild wapiti, bacterial and fungal functions were primarily associated with carbon metabolism and the pyruvate cycle, with mutualistic interactions prevailing among bacteria, fungi, and protozoa. Conversely, captive wapiti exhibited functional profiles centered on lipopolysaccharide and amino acid metabolism, also characterized by mutualistic coexistence among microbial communities. These findings highlight the significant impact of dietary composition on the gut microbiome. In summary, wild wapiti appear to possess a superior capacity for plant fiber utilization. These findings provide valuable data for the health management of farmed wapiti and their adaptability in natural habitats.

## Simple Summary

Diet and living environments can profoundly influence the composition of animal gut microbiota. Current research primarily focuses on detecting bacterial communities in animal intestines, with less attention paid to fungi and protozoa. This study comprehensively characterized bacteria, fungal and protozoan communities in fecal samples from wild and captive Chinese wapiti. Results revealed significant differences in gut microbial communities and functional characteristics between captive and wild wapiti. Fecal microbiota in captive wapiti was enriched with microbes involved in amino acid and fatty acid metabolism, exhibiting predominantly mutually exclusive interactions between bacteria, fungi, and protozoa. Conversely, wild wapiti harbored an abundant fecal microbiota associated with fiber utilization, characterized by largely mutualistic coexistence among bacteria, fungi, and protozoa. These findings suggest that domestication leads to alterations in the intestinal microbiota of wapiti.

## 1. Introduction

Diet stands as the primary determinant influencing and shaping the gastrointestinal microbiota of animals [1]. Owing to their distinct living environments, wild and domestic animals often consume different diets, which, in turn, affects the composition of their intestinal microbiota. For instance, studies have found that wild boars and domestic pigs harbor distinct dominant bacterial genera in their intestines due to dietary variations [2]. Similarly, wild yaks exhibit relatively abundant *Ruminococcaceae* and *Rikenellaceae*, which primarily participate in the degradation of fibrous substances, while domestic yaks have higher abundances of *Prevotellaceae*, *Alloprevotella*, and *Succinivibrio*, mainly involved in protein and carbohydrate degradation [3].

Deer, as ruminants, serve as crucial medical models for mammalian tissue and organ regeneration [4], and their digestive tract microbiota composition is also diet-dependent. The microbiota of grazing and captive reindeer may vary due to dietary differences [5]. Studies have shown that *Firmicutes* and *Bacteroides* are the predominant phyla in the digestive tracts of both wild and domestic deer. However, *Firmicutes* are more abundant in wild deer, while *Bacteroides* are more prevalent in domestic deer [6,7]. For instance, compared to wild sika deer, domestic sika deer exhibit a significant increase in *Fibrobacteres*, likely attributable to their high-fiber diets, such as hay [6]. In white-livened deer (*Cervus albirostris*), wild individuals have significantly higher abundances of *Firmicutes* and *Cyanobacteria* in their digestive tracts compared to domesticated ones, while *Spirochaetae*, *Bacteroides*, and *Verrucomicrobia* are lower [8]. Interestingly, in Red Deer (*Cervus elaphus*), captive herds have significantly lower levels of *Ruminococcaceae* than wild herds, possibly due to the low-fiber diet of captive red deer altering the fecal microbiota’s ability to degrade stubborn substrates like cellulose, hemicellulose, and lignocellulose [7]. These observations lead us to hypothesize that the diverse microbial structures serve distinct functions. However, previous studies have primarily focused on the compositional differences of digestive tract bacteria. Although bacteria are the most numerous microorganisms in the animal intestine and often dominate the intestinal microbiota [8,9,10,11], intestinal fungi, though fewer in number, secrete enzymes to break down lignin, cellulose, and hemicellulose, influencing pathogenic microorganism colonization [12,13]. Protozoa, the least reported group in the gut microbiome, especially in deer, often have symbiotic relationships with other microorganisms [14,15], influencing host immunity and intestinal homeostasis [16]. They also consume carbohydrates like cellulose and starch to provide energy for themselves and the host [17,18]. This knowledge gap significantly impedes our comprehensive understanding of the overall structure and function of the intestinal microbiota in deer.

Compared to common domestic animals such as pigs, cattle, and sheep, deer breeding technology remains relatively underdeveloped. As a unique livestock resource, wapiti are valued for their high-protein, low-fat meat products as well as regenerated antlers [19,20,21]. This study is the first to comprehensively characterize and compare the gut microbiota of wild and captive wapiti. The aim is to investigate the changes and evolutionary patterns of the intestinal flora in wapiti, providing crucial theoretical foundations and data support for the efficient breeding of domestic wapiti and the effective protection of wild wapiti.

## 2. Materials and Methods

### 2.1. Animals and Sample Collection

A total of 21 fecal samples were collected, including 10 wild wapiti samples from Gaogesitai Hanwula national nature reserve (Inner Mongolia Autonomous Region, China, 119°03′–119°39′ E, 44°41′–45°08′ N), and 11 captive deer samples from Baocheng Deer Industry Co. (Chifeng, Inner Mongolia Autonomous Region, China, 119°28′ E, 42°04′ N). To ensure the freshness of the samples, reduce environmental pollution, and avoid frightening the wild animals, we waited at the most frequented watering places of the deer herd to obtain fresh samples. Use disposable, sterile gloves to collect fresh captive wapiti droppings in the deer pen, minimizing disturbance to the animal’s activities as much as possible. All samples collected were carefully stripped to the surface using sterile forceps to preserve the internal feces, which were then preserved in sterile centrifuge tubes and immediately stored in liquid nitrogen to ensure that remained at low temperatures until sequencing was completed. All sample collections did not affect the normal activities and feeding habits of the animals.

### 2.2. Microbial Sequencing and Data Quality Control

Samples were extracted using the CTAB method, and PCR amplification was carried out using the samples as templates after their purity and concentration had been verified [19]. The specific primers for bacterial amplification were 341F (5′-CCTAYGGGRBGCASCAG-3′) and 806R (5′-GGACTACNNGGGTATCTAAT-3′), and the specific primers for archaeal amplification were 515F (5′-GTGCCAGCMGCCGCGGTAA-3′) and 806R (5′-GGACTACHVGGGTWTCTAAT-3′). The primers of fungi were ITS1F (5′-CTTGGTCATTTAGAGGAAGTAA’) and ITS2R (5′-GCTGCGTTCTTCATCGATGC-3′). 528F (5′-GCGGTAATTCCAGCTCCAA-3′) and 706R (5′-AATCCRAGAATTTCACCTCT-3′) for protozoa [20,21,22]. Library construction was performed using TruSeq^®^ DNA PCR-Free Sample Preparation Kit (Illumina, San Diego, CA, USA), and the constructed libraries were quantified by Qubit and Q-PCR, and after the libraries were qualified, they were used on NovaSeq6000 (Illumina, San Diego, CA, USA) for PE250 sequencing [23].

Paired-end reads were assigned to samples based on their unique barcode and truncated by cutting off the barcode and primer sequence. Paired-end reads were merged using FLASH (Version 1.2. 11, http://ccb.jhu.edu/software/FLASH/, accessed on 23 November 2025) [24], a very fast and accurate analysistool, which was designed to merge paired-end reads when at least some of the reads overlap theread generated from the opposite end of the same DNA fragment, and the splicing sequences were called raw tags. The spliced Raw Tags were strictly filtered by fastp software (Version 0.23.1) to obtain high-quality Tags data [25]. The Tags obtained after the above processing need to be processed to remove chimeric sequences, and the Tags sequences were compared with the species annotation database (Silva database (16S/18S), https://www.arb-silva.de/, accessed on 23 November 2025; Unite Database (ITS), https://unite.ut.ee/, accessed on 23 November 2025) to detect chimeric sequences, and the chimeric sequences were finally removed to obtain the final Effective Tag [26]. For the Effective Tags obtained previously, denoise was performed with DADA2 or deblur module in the QIIME2 software (Version QIIME2-202202) to obtain initial ASVs (Amplicon Sequence Variants) (default: DADA2) [27]. Species annotation was performed using QIIME2 software. For 16S/18S, the annotation database is Silva Database, while for ITS, it is Unite Database. The absolute abundance of ASVs was normalized using a standard of sequence number corresponding to the sample with the least sequences.

### 2.3. Data Analysis and Visualization

To ensure the reliability of downstream analysis, we evaluated the sequencing depth. The Rarefaction curves calculated based on the number of effective Tags have all reached the plateau period, indicating that the current sequencing depth is sufficient to cover the vast majority of microbial diversity in the samples. Subsequently, the Alpha diversity index of each sample was calculated using QIIME2 software to assess the microbial diversity within the samples. The Wilcoxon test was used to analyze the significant differences in the microbial α-diversity index between the DA group and the WA group. The Metastat analysis using RStudio software (4.5.1) (with automatic FDR correction) was used to calculate the significant differences in the genus levels of bacteria, fungi and protists between the DA group and the WA group. The microeco and ggplot2 of the RStudio software were used to calculate and plot the horizontal stacking maps and α diversity indices of bacterial, fungal and protozoan phyla. The functional characteristics of fecal bacteria were predicted by Tax4Fun2, and those of fecal fungi were predicted by PICRUSt2 (v2.6.0) [28]. And the Wilcoxon test was used to analyze the significant differences in metabolic pathways at Level3 between bacteria and fungi. Based on the weighted UniFrac distance and the Bray–Curtis dissimilarity matrix, microbial communities and functions between groups were compared using Principal coordinates analysis (PCoA). Principal component analysis (PCA) was calculated by the prcomp function of the stats package in the RStudio software and the ropls package. Both R^2^ and *p* values were analyzed and calculated using the Adonis function from the vegan package, which conducts permutation multivariate analysis of variance [29]. The stats package of the RStudio software is used to calculate the Spearman rank correlation coefficient with thresholds of *p* < 0.05 and R > 0.6 or <−0.6, and the Gephi software (0.10.1) is used to visualize the correlation network [30].

## 3. Results

### 3.1. Compositional Characteristics and Predicted Metagenomic Functions in Bacteria

**Figure 1:**
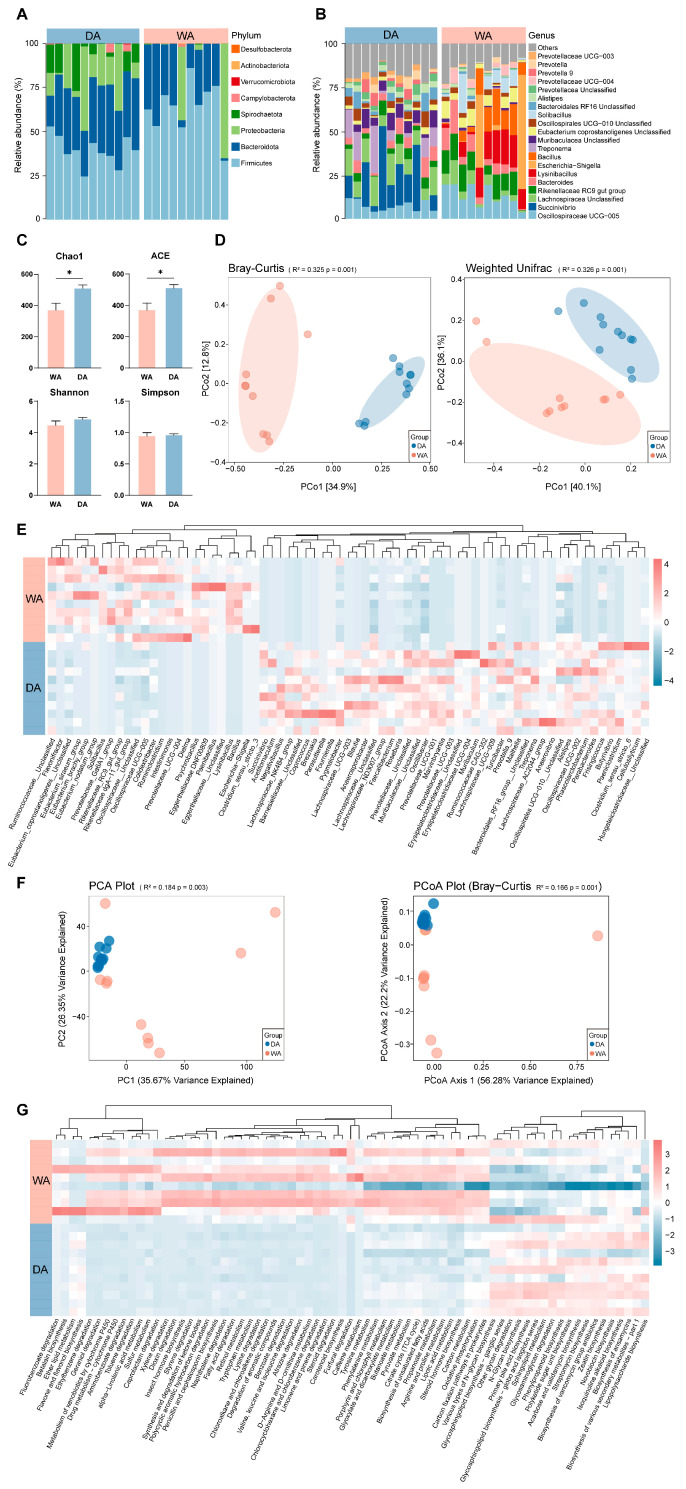
Composition, diversity and functional prediction of bacterial microbial community in the feces of wild (WA) and captive (DA) wapiti. (**A**) Microbial composition at the phylum level of the intestinal bacteria of wapiti in the WA and DA groups. (**B**) Bacterial composition at the genus level of the intestinal bacteria of wapiti in the WA and the DA groups. (**C**) Comparison of α diversity index between the WA and DA groups. ACE: Abundance-based Coverage Estimator. (**D**) PCoA (principal co-ordinates analysis) plots based on the Bray–Curtis and Weighted Unifrac algorithms describing the differences in microbial community composition and structure at the OTU level in feces of wapiti between the WA and DA groups. (**E**) Heat map describing the significant differences in microbial composition between the WA and DA groups (*p* < 0.05). (**F**) PCoA plot of the differences in bacterial functional characteristics at the KEGG (Kyoto Encyclopedia of Genes and Genomes) 3 level (relative abundance) based on Bray–Curtis dissimilarity. (**G**) Description of the significant difference pathways of KEGG 3 between the WA and DA groups (*p* < 0.05). * *p* < 0.05.

After denoising and species annotation of the sample sequencing data using the QIIME2 software package, a total of 1,486,121 reads were retained and classified into 6769 OTUs (Table S1). Further classification identified 19 phyla, with *Firmicutes* (DA, 41.75%; WA, 59.72%), *Bacteroidota* (DA, 35.42%; WA, 21.23%), and *Proteobacteria* (DA, 13.82%; WA, 17.25%) dominat phyla (Figure 1A). Thirty-five genera were further confirmed, with the dominant genera differing between the two groups. The relative abundance of *Escherichia-Shigella* (DA, 0.00%; WA, 9.69%), *UCG-005* (DA, 6.18%; WA, 9.19%), and *Rikenellaceae-RC9-gut-group* (DA, 5.54%; WA, 7.03%) was relatively higher in the WA group. While the relative abuntivities of *Succinivibrio* (DA, 13.57%; WA, 0.00%) and *Treponema* (DA, 7.14%; WA, 0.00%) were higher in the DA group (Figure 1B). Analysis of the α-diversity index of the WA group and the DA group indices between the WA and DA groups indicated that the Chao1 index and ACE index were significantly increased in the DA group (*p* < 0.05), while the other indicators did not differ significantly (*p* > 0.05) (Figure 1C). Bray–Curtis and Weighted Unifrac analyses revealed a significant difference in the gut microbiome composition between wild and captive wapiti (*p* = 0.001) (Figure 1D). The abundance of *Bacillus* (DA, 0.00%; WA, 5.50%), *Lysinibacillus* (DA, 0.00%; WA, 6.81%), and *Ruminiclostridium* (DA, 0.00%; WA, 0.03%) were significantly higher in the WA group than those in the DA group. The abundance of *Negativibacillus* (DA, 0.04%; WA, 0.02%), *Fournierella* (DA, 0.16%; WA, 0.00%), *Blautia* (DA, 0.46%; WA, 0.08%), *Anaerosporobacter* (DA, 0.62%; WA, 0.00%), *Unclassified__Lachnospiraceae* (DA, 0.01%; WA, 0.00%), *Prevotellaceae_UCG-003* (DA, 1.97%; WA, 0.16%), *Prevotella_9* (DA, 2.29%; WA, 0.01%), and *Clostridium_sensu_stricto_6* (DA, 0.12%; WA, 0.00%) were significantly higher in the DA group than in the WA group (*p* < 0.05) (Figure 1E). We applied Tax4Fun2 to predict the potential functions of intestinal bacteria in wapiti and compared the differences between the two groups. PCoA results indicated significant differences in metabolic pathways at the KEGG 3 level between the WA and DA groups (Figure 1F) (*p* < 0.01). In addition, significant differences were observed in 71 metabolic pathways between the WA and DA groups (*p* < 0.05). The WA group showed a significant positive correlation in pathways related to signal transduction and material circulation, including carbon metabolism, Pyruvate metabolism, steroid hormone biosynthesis, and Citrate cycle (TCA cycle) (*p* < 0.05). While pathways related to biomolecule anabolism, including lipopolysaccharide biosynthesis, glycosaminoglycan degradation, and Primary bile acid biosynthesis, showed a significant significantly negative correlation (*p* < 0.05) (Figure 1G).

### 3.2. Compositional Characteristics and Predicted Metagenomic Functions in Fungi

**Figure 2:**
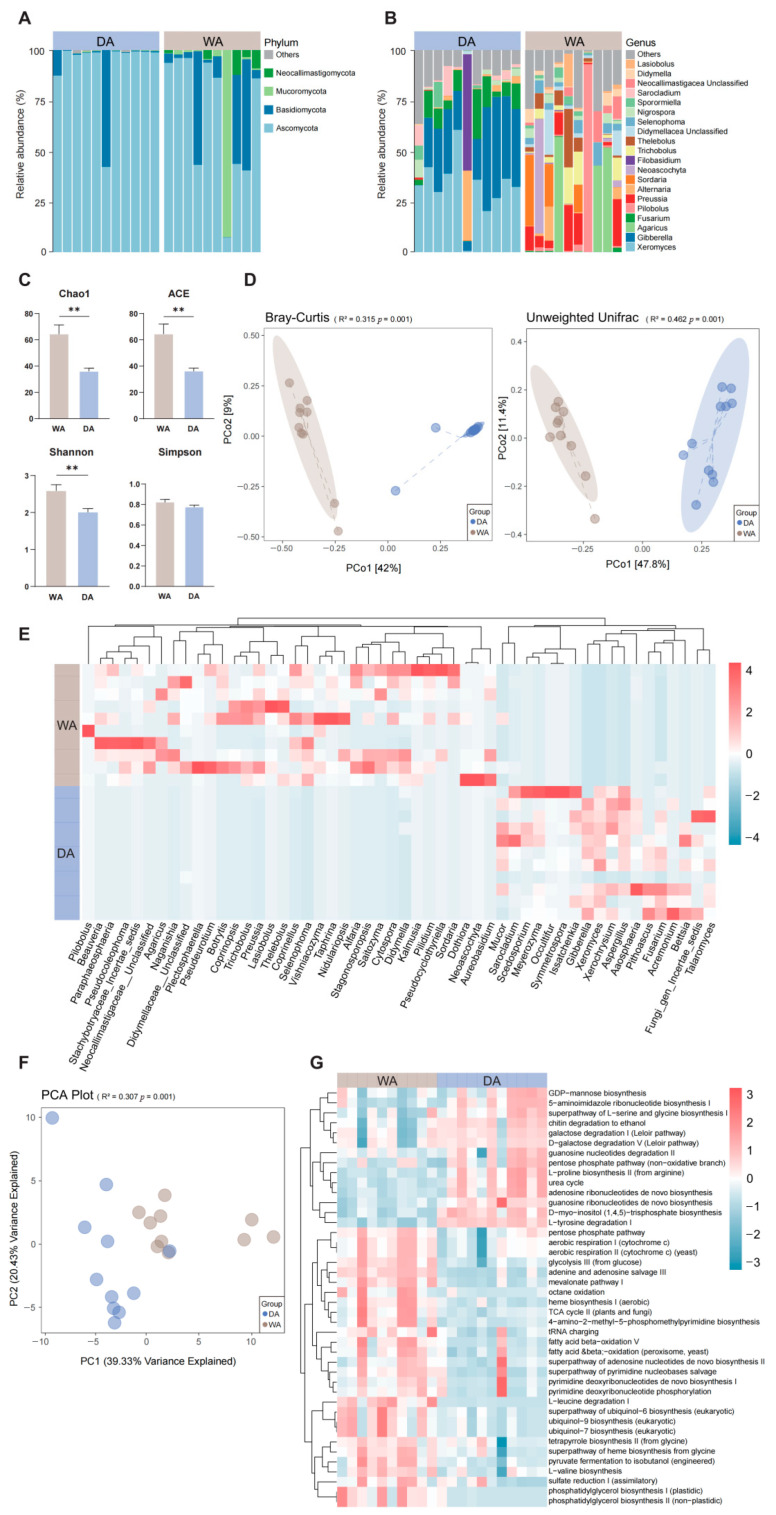
Composition, diversity and functional prediction of fungal microbial community in the feces of wild (WA) and captive (DA) wapiti. (**A**) Microbial composition at the fungal phylum level in the intestinal tract of wapiti in the WA and DA groups. (**B**) Microbial composition at the fungal genus level in the intestinal tract of wapiti in the WA and DA groups. (**C**) Comparison of α diversity index between the WA and DA groups. ACE: Abundance-based Coverage Estimator. (**D**) PCoA (principal co-ordinates analysis) plots based on the Bray–Curtis and Unweighted Unifrac algorithms describing the e differences in the members and structure of the fungal microbial community in the feces of wapiti between the WA and DA groups at the OTU level; (**E**) Heat map describing the significant differences in microbial composition between the WA group and the DA group (*p* < 0.05); (**F**) PCA graph based on Bray–Curtis dissimilarity; (**G**) KEGG (Kyoto Encyclopedia of Genes and Genomes) 3 pathways describing the significant differences of between the WA group and the DA group (*p* < 0.05). ** *p* < 0.01.

After denoising and species annotation of the sample sequencing data using the QIIME 2 software package, a total of 956,719 reads were retained and classified into 2114 OTUs (Table S2). Further classification identified 15 phyla, with *Ascomycota* (DA, 63.43%; WA, 41.11%) and *Basidiomycota* (DA, 3.29%; WA, 16.53%) being the predominant phyla in both groups. In the WA group, the relative abundances of *Mucoromycota* (DA, 0.32%; WA, 8.08%) and *Neocallimastigomycota* (DA, 0.95%; WA, 1.29%) increased (Figure 2A), and a further 35 genera were identified. In the WA group, *Agaricus* (DA, 0.00%; WA, 7.47%), *Preussia* (DA, 0.00%; WA, 5.45%), and *Thelebolus* (DA, 0.00%; WA, 3.86%) was more abundant. In the DA group, *Xeromyces* (DA, 20.29%; WA, 0.00%), *Gibberella* (DA, 18.84%; WA, 0.00%), and *Fusarium* (DA, 6.54%; WA, 0.00%) were more abundant, indicating a more complex fungal microbiome in the WA group (Figure 2B). Analysis of α-diversity indices between the WA and the DA groups indicated that the Chao1 index, ACE index, and Shannon index of the DA group increased significantly (*p* < 0.05), while the differences in other indices were not statistically significant (*p* > 0.05) (Figure 2C). The gut fungal composition of wild and captive wapiti was significantly different (*p* = 0.001) according to Bray–Curtis and Unweighted Unifrac analyses (Figure 2D). The abundances of *Beauveria* (DA, 0.00%; WA, 0.32%), *Paraphaeosphaeria* (DA, 0.00%; WA, 0.61%), *Naganishia* (DA, 0.00%; WA, 0.07%), and *Selenophoma* (DA, 0.00%; WA, 1.64%) in the WA group were significantly higher than those in the DA group (*p* < 0.05), while the abundances of *Mucor* (DA, 0.31%; WA, 0.17%), *Acremonium* (DA, 0.05%; WA, 0.00%), *Fungi_gen_Incertae_sedis* (DA, 1.59%; WA, 0.81%), and *Bettsia* (DA, 0.05%; WA, 0.00%) in the DA group were significantly higher than those in the WA group (*p* < 0.05) (Figure 2E). When predicting the potential functions of the intestinal microbiota of wapiti, significant differences in metabolic pathways at the KEGG 3-level were found between the WA and DA groups (*p* < 0.01) (Figure 2F). In addition, there were significant differences in 42 metabolic pathways between the WA group and the DA group (*p* < 0.05). The signal transduction and material circulation-related pathways in the WA group, including glycolysis III (from glucose), TCA cycle II (plants and fungi), aerobic respiration I (cytochrome c), aerobic respiration II (cytochrome c) (yeast), were significantly positive correlated (*p* < 0.05). In contrast, pathways related to biomolecular synthesis, including the superpathway of L-serine and glycine, L-proline biosynthesis II (from arginine), urea cycle, pentose phosphate pathway (non-oxidative branch), and D-myo-inositol (1,4,5)-trisphosphate biosynthesis, were significantly negative correlated (*p* < 0.05) (Figure 2G).

### 3.3. Exploration the Protozoa Composition in the Gut of Wapiti

**Figure 3:**
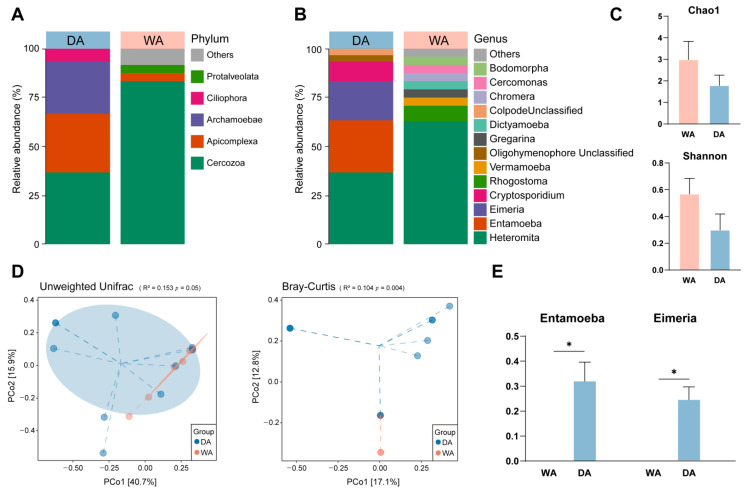
Composition and diversity of protozoan microbial communities in the feces of wild (WA) and captive (DA) wapiti. (**A**) Phylum-level composition of protozoa in the intestines of wapiti in the WA and DA groups; (**B**) Genus-level composition of protozoa in the intestines of wapiti in the WA and DA groups; (**C**) Comparison of α diversity index between the WA and DA groups; (**D**) PCoA (principal co-ordinates analysis) plots showing differences in protozoan microbial communities composition and structure at the OTU level in the feces of wapiti between the WA and DA groups, based on the Bray–Curtis and Unweighted Unifrac algorithms, respectively; (**E**) Bar chart showing significant differences in microbial composition between the WA and DA groups (*p* < 0.05).* *p* < 0.05.

We sought to explore and characterize the presence of protozoa in the intestines of wild and captive wapiti. The noise reduction and annotation of protozoan ASV depended on the pre-analysis removal of chloroplasts and fungi. After quality control. A total of 3726 reads were retained and classified into 41 OTUs (Table S3). At the phylum level, we found that *Cercozoa* (DA, 33.33%; WA, 66.67%), *Apicomplexa* (DA, 27.27%; WA, 0.00%), *Archamoebae* (DA, 24.24%; WA, 3.33%), and *Ciliophora* (DA, 6.06%; WA, 0.00%) are the dominant phyla in the DA group. While *Cercozoa*, *Apicomplexa*, *unidentified_Amoebozoa* (DA, 0.00%; WA, 6.67%) and *Protalveolata* (DA, 0.00%; WA, 3.33%) are the dominant phyla in the WA group (Figure 3A). The dominant bacterial genera in the two groups differed. In the WA group, *Heteromita* (DA, 33.33%; WA, 50.00%), *Rhogostoma* (DA, 0.00%; WA, 6.67%), *Vermamoeba* (DA, 0.00%; WA, 3.33%), and *Gregarina* (DA, 0.00%; WA, 3.33%) was relatively abundant. While in the DA group, soil flagellates such as *Heteromita*, *Entamoeba* (DA, 24.24%; WA, 0.00%), *Eimeria* (DA, 18.18%; WA, 0.00%), and *Cryptosporidium* (DA, 9.09%; WA, 0.00%) were relatively abundant, and the protozoan composition in the WA group was more complex (Figure 3B). Genus-level analysis revealed significantly higher abundance of *Entamoeba* and *Eimeria* in the DA group compared with the WA group (*p* < 0.05) (Figure 3E). Alpha-diversity index analysis revealed no significant differences in the Chao1 index or Shannon index between the WA and DA groups (*p* > 0.05) (Figure 3C). Bray–Curtis analysis revealed highly significant differences in the intestinal protozoa composition between wild and captive wapiti (*p* < 0.01) (Figure 3D).

### 3.4. Interaction Analysis of Bacterial, Fungal, and Protozoan Microbial Networks Between Wild and Captive Wapiti

**Figure 4:**
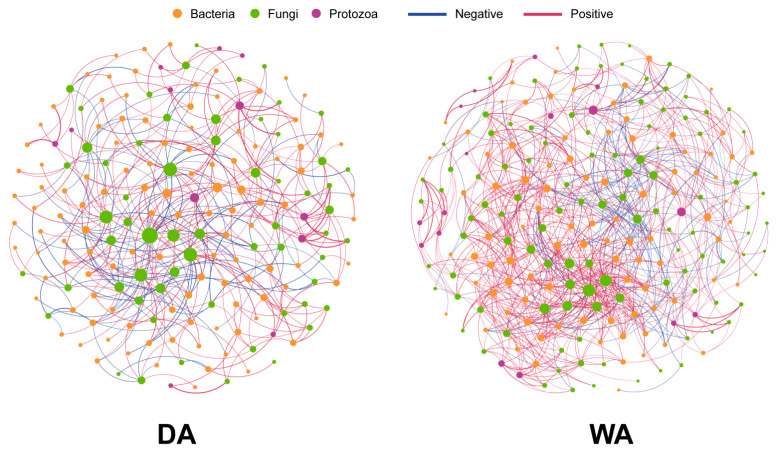
Spearman correlation analysis of bacterial, fungal, and protozoan microbial interaction networks between wild and captive wapiti. The pink and blue lines indicate positive and negative correlations, respectively.

We respectively studied the correlations between the altered microorganisms in the WA group and the DA group (Figure 4). The results revealed that the co-occurrence network for the WA group comprised 141 nodes and 326 edge data points, whereas that for the DA group contained 123 nodes and 218 edge data points (Table S4). Notably, within the WA group, 234 edges representing positive correlations and 83 edges indicating negative correlations were observed in the fungal-bacterial edge data, whereas the fungal-protozoan edge data exhibited 9 positive correlations and 0 negative correlations (Table S5). Within the DA group, fungal-bacterial edge data comprised 91 edges indicating positive correlations and 100 edges indicating negative correlations. Fungal-protozoan edge data comprised 22 edges indicating positive correlations and 5 edges indicating negative correlations (Table S6). Within the WA group, *Agaricus* exhibits negative correlations with *Escherichia-Shigella* and positive correlations with *Ruminococcus*, and *Hungateiclostridiaceae__Unclassified*. *Preussia* shows positive correlations with *Clostridium_sensu_stricto_3*, and *Eggerthellaceae__Unclassified*, while *Prevotellaceae_UCG-004* and *Rikenellaceae_dgA-11_gut_group* showed negative correlations (Table S5). Within the DA group, *Xeromyces* exhibited negative correlations with *Prevotellaceae__Unclassified* and *Papillibacter. Gibberella* showed a negative correlation with *Oscillospiraceae_NK4A214_group*, and *Fusarium* demonstrated a positive correlation with *Anaerovibrio* (Table S6).

## 4. Discussion

Previous studies have demonstrated significant differences in fecal microbial communities between wild and captive reindeer, a finding mirrored in our research on wild and captive wapiti [8]. At the phylum level, *Bacteroidetes* and *Firmicutes* dominate the digestive tracts of wapiti in Xinjiang [9], suggesting regional similarity in their gut microbiota composition. Wild sika deer exhibit a higher prevalence of *Firmicutes* compared to their domesticated counterparts, whereas *Bacteroidetes* and *Proteobacteria* are more prevalent in domesticated sika deer [6], aligning with our study. *Bacteroidetes* facilitate the degradation of monosaccharides, proteins, and carbohydrates, whilst Firmicutes primarily break down cellulose [3,31]. Wild red deer consume untreated, high-fiber natural diets, necessitating a greater presence of fiber-degrading bacteria [32]. In contrast, domesticated animal feed typically contains lower fiber levels and simpler carbohydrates [33], explaining the observed compositional shift. Furthermore, dominant bacterial genera differed between groups. Wild wapiti had higher levels of *Escherichia-Shigella* and *UCG-005*. *Escherichia-Shigella*, a marker for intestinal inflammation in animals [34], has been linked to diarrhea in various species [35] and detected in wild animals like the Alpine musk deer and giant panda [36,37]. Its abundance is subject to seasonal fluctuations, increasing notably in summer [38,39]. Our sampling during the summer-autumn transition suggests seasonal influences should be taken into account. *UCG-005*, a butyrate-producing bacterium involved in cellulose degradation, converts cellulose into glucose and fructose, which undergo glycolysis to form pyruvate, entering the tricarboxylic acid cycle to generate energy [40,41,42,43]. Research indicates that the relative abundance of propionate metabolic pathways in wild reindeer is significantly higher than in captive reindeer [5], consistent with our findings. These pathways are associated with cellulose degradation and energy metabolism [44], indicating enhanced plant fiber utilization capacity in wild wapiti. Conversely, domesticated wapiti exhibited higher relative abundances of *Succinivibrio* and *Treponema*. *Treponema*, a potential pathogen [45], has unconfirmed pathogenicity in animal gastrointestinal tracts [46,47]. Xue et al. [48] identified *Succinivibrio* as a common short-chain fatty acid producer in ruminant intestines, with its abundance positively correlated with acetate, propionate, and butyrate levels, which serve growth, development, and energy metabolism. Beyond these abundant genera, *Clostridium_sensu_stricto_6* was more prevalent in captive wapiti, potentially participating in amino acid utilization through bile acid metabolism [5], consistent with the enrichment of bile acid metabolic pathways observed in this study. Grain-fed captive wapiti often have high fat and amino acid levels, similar to domestic donkeys whose gut microbiota enriches lipopolysaccharide-associated metabolic pathways [49], aligning with the present findings. However, predictive accuracy depends on database availability and phylogenetic distance from reference genomes [7], necessitating further validation through multi-omics studies (e.g., transcriptomics, metabolomics).

Ruminant gut fungi possess a rich array of carbohydrate-hydrolyzing enzymes, including β-glucosidases and xylanases, crucial for herbivores [50,51,52]. Previous studies have identified Ascomycota and Basidiomycota as predominant fungal phyla in ruminant gastrointestinal tracts [53,54], which is consistent with the digestive tract fungal composition observed in wapiti in the digestive tract of this study. An increased *Basidiomycota* to *Ascomycota* ratio may contribute to fungal microbiota dysbiosis and inflammatory bowel disease [16]. Our findings indicate that wild wapiti exhibit higher proportions, suggesting a potential risk of intestinal inflammation within this population. Furthermore, the proportions of *Basidiomycota* and *Ascomycota* differ among various animal species, which may be influenced by factors such as their habitat, diet, and the structure of their intestinal microecology [55]. Compared to captive wapiti, wild ones displayed greater relative abundances of *Mucoromycota*, *Neocallimastigomycota*, *Agaricus*, and *Preussia*. *Mucoromycota*, frequently found in soil [53], may have been ingested during foraging. *Neocallimastigomycota*, rich in cellulase and hemicellulase, efficiently degrade plant cell wall components into fermentable monosaccharides, supporting high-quality microbial protein production for the host [56,57], aligning with wild wapiti’s dietary characteristics. Although *Agaricus* exhibits relatively high abundance in the digestive tracts of wild wapiti, it does not appear to be a common fungal inhabitant of animal intestines, with only a few studies reporting its presence [58,59]. However, due to the absence of comprehensive data on the wild wapiti’s diet, we hypothesize that the observed material may stem from undigested plant residues following foraging activities, rather than indicating a long-term colonization within the gut of wild wapiti [60]. *Preussia*, currently identified only in plant material and African savanna elephant feces [61,62,63], possesses the capacity to produce cellulase, amylase, or lipase [63,64,65]. Conversely, the specific functions and roles of certain fungi within animal intestines remain ambiguous, necessitating further exploration. Notably, the enrichment of cellulose-degrading fungi resembles the bacterial functional outcomes observed in the digestive tracts of wild wapiti; wild wapiti mirrors the bacterial functional profiles observed in their digestive tracts. Conversely, captive wapiti exhibit higher relative abundances of *Xeromyces*, *Gibberella*, and *Fusarium*. *Xeromyces*, a drought-tolerant extremophile fungus [66], is prevalent in the intestines and feces of various animals and shows a significant negative correlation with acetic acid production [67]. It is also associated with diseases such as depression and hypertension [68,69], and can influence the secretion of the host’s anti-inflammatory cytokine IL-4 [70]. For wapiti, *Gibberella* is an exogenous intestinal fungus and a common plant pathogen found in cereals like maize [71], often accompanied by *Fusarium* [72]. Given that maize and maize stover are primary roughage sources for captive wapiti, the inferior quality of roughage provided by breeders likely contributes to the proliferation of these phytopathogenic fungi [73]. However, this does not imply that there is a significant presence of pathogenic bacteria in the intestines of captive wapiti. This may be attributed to the fact that we collected samples from captive wapiti at only one farm and the sample size was limited, which constrained the generalizability of our findings. Functional predictions reveal that gut fungi in wild wapiti enrich pathways linked to glycolysis, the TCA cycle, and aerobic respiration, all crucial for fiber utilization [42,74]. In contrast, the gut microbiota of captive wapiti show enrichment in pathways related to L-serine and glycine, L-proline biosynthesis II (from arginine), and the urea cycle, which are involved in amino acid and lipid metabolism [75,76]. This pattern mirrors the functional predictions of gut bacteria in both wild and captive wapiti.

Protozoa, particularly ciliates, are intriguing microorganisms. Soil protozoa have been demonstrated to function as bacterial predators and fungal nurturers, among other roles [77]. They typically form symbiotic relationships with their hosts and exhibit co-evolutionary patterns during long-term ecological succession [78,79]. In this study, *Heteromita* emerged as the dominant genus in the digestive tracts of wild wapiti. *Heteromita* closely interacts with bacteria, aiding in toluene degradation and hydrocarbon breakdown [80]. Despite being a dominant genus in both wild and captive wapiti, its specific functions within the animal’s digestive tract remain underexplored and warrant further investigation. In contrast, *Entamoeba* and *Eimeria* are more abundant in the digestive tracts of captive wapiti. These protozoa are commonly found in animal intestines and may possess pathogenic potential [81,82]. These findings suggest that monitoring animal health should not be limited to bacterial composition alone. However, limitations in sample size and sequencing methods may have led to the underdetection of numerous protozoa.

Research underscores the crucial roles of complex interactions among bacteria, fungi, and protozoa in the development, growth, adaptation, and diversification of organisms [83,84]. Zou et al. [55] observed that gut bacterial and fungal communities in wild herbivores tend towards coexistence rather than mutual exclusion. Conversely, in captive herbivores, these communities exhibit a tendency towards mutual exclusion [85]. Studies indicate that dietary alterations can rapidly induce shifts in gut microbiota and alter intergenic relationships [86,87]. Research on artificially fed Indian camels (Camelus dromedarius) revealed predominantly negative correlations between gastrointestinal fungi and protozoa [88], consistent with our findings. However, this may also result from the influence of antibiotics or biological control agents in captive animals [89]. Factors such as sex may further modulate microbial interactions [90]. This highlights that microbial interactions are influenced by multiple factors, including diet and sex, necessitating further research to resolve these discrepancies and elucidate the contextual drivers of microbial variation. At the specific microbial interaction level, dominant genera in wild wapiti—*Agaricus* and *Preussia*—exhibit positive correlations with cellulose-degrading bacteria (*Ruminococcus* and *Clostridium_sensu_stricto_3*) [91,92]. Simultaneously, they suppress potential pathogenic bacteria *Escherichia-Shigella* [34], forming a synergistic coexistence conducive to fiber degradation. Conversely, the core fungi *Xeromyces* in captive wapiti show negative correlations with primary fiber-degrading bacteria of the *Prevotellaceae* family [93] and lipid-regulating bacteria *Papillibacter* [94]. This mutually exclusive development aligns with the bacterial-fungal interactions observed in captive animals.

Our findings reveal distinct microbial structures between captive and wild wapiti, offering valuable insights for wapiti herders, given the gut microbiota’s significance for animal health and productivity [95]. For national nature reserves, providing health monitoring data on wild wapiti is also crucial. However, this study has limitations: findings were derived from a limited sample size of wapiti, highlighting the need for further research. Additionally, wild wapiti samples did not account for sex or age factors, while captive wapiti samples were collected from a single breeding farm, potentially limiting the generalizability of the results. Future research should address these limitations by comparing gut microbial compositions between female and male wapiti and across different seasons, incorporating larger and more diverse samples, and employing methodologies like transcriptomics and metabolomics.

## 5. Conclusions

We conducted comparative analyses of bacteria, fungi, and protozoa in wild and captive wapiti, revealing significant differences in gut microbiota composition between wild and captive wapiti. The microbial communities in wild wapiti feces were associated with cellulose degradation, whereas those in captive wapiti feces were linked to amino acid and fatty acid utilization. These findings highlight the influence of dietary composition on the gut microbiome and may provide new insights into the husbandry management of captive wapiti, ultimately leading to efficient, scientific, and healthy rearing practices.

## Data Availability

Sequence data that support the findings of this study have been deposited in the National Center for Biotechnology Information: https://www.ncbi.nlm.nih.gov, accessed on 23 November 2025, PRJNA 1172148, 1172087, 1172100, 1172092, 1172002, 1172008, 1171993 and 1168658. The original contributions presented in the study are included in the article, further inquiries can be directed to the corresponding author.

## Supplementary Materials

The following supporting information can be downloaded at: https://www.mdpi.com/article/10.3390/ani16010044/s1, Table S1. Sequencing depth of fecal bacteria of wild and captive wapiti; Table S2. Sequencing depth of fecal fungi of wild and captive wapiti; Table S3. Sequencing depth of fecal protozoa of wild and captive wapiti; Table S4. The co-occurrence network parameter of wild and captive wild; Table S5. The correlation of bacterial and fungal microbiomes in the feces of wild wapiti; Table S6. The correlation of bacterial and fungal microbiomes in the feces of captive wapiti.
